# Light‐Driven WSe_2_‐ZnO Junction Field‐Effect Transistors for High‐Performance Photodetection

**DOI:** 10.1002/advs.201901637

**Published:** 2019-11-11

**Authors:** Nan Guo, Lin Xiao, Fan Gong, Man Luo, Fang Wang, Yi Jia, Huicong Chang, Junku Liu, Qing Li, Yang Wu, Yang Wang, Chongxin Shan, Yang Xu, Peng Zhou, Weida Hu

**Affiliations:** ^1^ Qian Xuesen Laboratory of Space Technology China Academy of Space Technology Beijing 100094 China; ^2^ State Key Laboratory of Infrared Physics Shanghai Institute of Technical Physics Chinese Academy of Sciences 500 Yutian Road Shanghai 200083 China; ^3^ Jiangsu Key Laboratory of ASIC Design Nantong University Nantong Jiangsu 226019 China; ^4^ Hangzhou Institute for Advanced Study University of Chinese Academy of Sciences Hangzhou 310024 China; ^5^ Department of Physics and Tsinghua‐Foxconn Nanotechnology Research Center Tsinghua University Beijing 100084 China; ^6^ Henan Key Laboratory of Diamond Optoelectronic Materials and Devices School of Physics and Engineering Zhengzhou University Zhengzhou 450001 China; ^7^ School of Information Science and Electronic Engineering College of Microelectronics Zhejiang University Hangzhou 310027 China; ^8^ State Key Laboratory of ASIC and System Department of Microelectronics Fudan University Shanghai 200433 China

**Keywords:** gain, junction field‐effect transistors, photoresponse, response time, tradeoff

## Abstract

Assembling nanomaterials into hybrid structures provides a promising and flexible route to reach ultrahigh responsivity by introducing a trap‐assisted gain (*G*) mechanism. However, the high‐gain photodetectors benefitting from long carrier lifetime often possess slow response time (*t*) due to the inherent *G*–*t* tradeoff. Here, a light‐driven junction field‐effect transistor (LJFET), consisting of an n‐type ZnO belt as the channel material and a p‐type WSe_2_ nanosheet as a photoactive gate material, to break the *G*–*t* tradeoff through decoupling the gain from carrier lifetime is reported. The photoactive gate material WSe_2_ under illumination enables a conductive path for externally applied voltage, which modulates the depletion region within the ZnO channel efficiently. The gain and response time are separately determined by the field effect modulation and the switching speed of LJFET. As a result, a high responsivity of 4.83 × 10^3^ A W^−1^ with a gain of ≈10^4^ and a rapid response time of ≈10 µs are obtained simultaneously. The LJFET architecture offers a new approach to realize high‐gain and fast‐response photodetectors without the *G*–*t* tradeoff.

## Graphical abstract

The concept of a light‐driven junction field‐effect transistor (LJFET) is proposed to break the gain‐response time tradeoff for high‐gain phototransistors. The gain and response time are separately determined by the field effect modulation and the switching speed of LJFET. The design offers a new approach for photodetectors to reach high gain and fast response simultaneously.

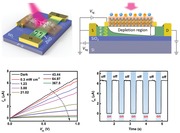

Nanomaterials have shown great potential as photosensitive elements thanks to their unique properties, such as broadband response and fast response time.^1^, ^2^, ^3^, ^4^, ^5^, ^6^, ^7^, ^8^, ^9^ After years of study on the photoresponse of single‐material‐based devices, researchers have begun to combine different nanomaterials into hybrid structures in order to enhance the performance of photodetectors.^10^, ^11^, ^12^, ^13^, ^14^, ^15^, ^16^, ^17^ Especially, employing sensitizers to decorate the transport channels has been widely adopted to achieve an ultrahigh gain based on photogating effect.^18^, ^19^, ^20^, ^21^, ^22^, ^23^, ^24^, ^25^, ^26^ However, one obstacle in such devices is that the long lifetime of photoexcited carriers captured in sensitizers would result in slow response, which is a common problem with trap‐assisted gain mechanism. Though great efforts have been made to use various materials or device structures to regulate the gain–response time (*G*–*t*) tradeoff,^27^, ^28^, ^29^ the key to solving this issue is how to decouple the gain from carrier lifetime. In addition, strongly light‐absorbing materials, such as colloidal quantum dots (CQDs), have been widely used in high‐gain phototransistors (2D material‐CQDs phototransistors^18^, ^20^, ^21^, ^22^, ^27^ and thin‐film semiconducting material‐CQDs phototransistors^30^, ^31^) in order to enhance the photogate. The performance of these devices largely depends on the light absorption capacity of sensitizer. This restricts the versatility in realizing high‐gain photodetectors in other materials.

In this work, a light‐driven junction field‐effect transistor (LJFET) is proposed to overcome the *G*–*t* tradeoff to achieve high‐performance photodetection. The LJFET configuration contains an n‐type ZnO belt as the channel material on which is a p‐type WSe_2_ nanosheet as a photoactive top‐gate material. As it shows below, the WSe_2_ works as a light‐driven switch to control the application of top‐gate voltage on the ZnO channel. As a result, the channel conductance is modulated by externally applied top‐gate voltage rather than photogate or photovoltage induced by sensitizers, and the response time is determined by the switching speed of LJFET. The experimental results exhibit a high responsivity of 4.83 × 10^3^ A W^−1^ with a gain of ≈10^4^ and a fast response time of ≈10 µs at the same time. Moreover, because the bandgap of WSe_2_ nanosheet is ≈1.2 eV,^32^ the photoresponse covers a spectral range from the visible to near‐infrared.

**Figure** **1**a shows an illustration of the WSe_2_‐ZnO LJFET. In this architecture, WSe_2_ nanosheet has two key functions: 1) to form a p–n junction with n‐type ZnO and 2) to be a photoactive gate material between the side top‐gate electrode (TG) and the junction. In the dark, the resistance of WSe_2_ is high enough to weaken the effect of top‐gate voltage (*V*_tg_) on the ZnO channel. The depth of depletion region within ZnO channel is hard to be affected (Figure 1b). Under light illumination, when the photon energy is chosen between the bandgap of ZnO (≈3.3 eV)^33^, ^34^ and that of WSe_2_ (≈1.2 eV), only photocarriers in WSe_2_ are excited (Figure 1c). The reduced resistance of WSe_2_ enables a conductive path for the negative *V*_tg_ that reversely biases the junction. The enlarged depletion region within ZnO leads to a dramatic increase in the channel resistance (Figure 1d). As discussed below, the positive *V*_tg_ as a forward bias will shrink the depletion region, leading to a decrease in the channel resistance. Figure 1e,f presents the simulated electron density distribution of LJFET. A large decrease in electron density of ZnO channel can be observed with illumination. It is worth noting that the illumination also induces a photovoltage at the junction due to the photovoltaic effect (same as the open‐circuit voltage of solar cells). In order to clarify these two mechanisms, we also simulate WSe_2_‐ZnO photovoltage field‐effect transistor (PVFET) as shown in Figure S1 (Supporting Information). The photocarriers in WSe_2_ produce a photovoltage. This weak forward bias shrinks the depletion region slightly and induces a small increase in electron density. Though transition metal dichalcogenides are demonstrated to have strong light‐matter interactions^35^ and can be adopted as sensitizers,^36^, ^37^ the WSe_2_ nanosheet used in our device only induces a weak photovoltage at WSe_2_‐ZnO junction under light excitation. Apparently, *V*_tg_ dominates the photoresponse for the WSe_2_‐ZnO LJFET. This is in agreement with experiment results which will be discussed below in detail.

**Figure 1:**
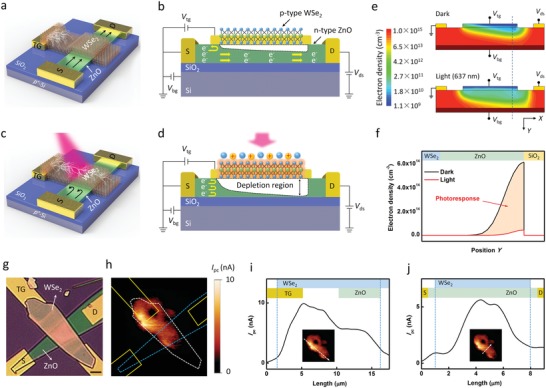
Structure and operating mechanism of WSe_2_‐ZnO LJFET. a) A schematic illustration of the device in the dark. The p‐type WSe_2_ nanosheet as the gate material is transferred onto the n‐type ZnO channel and side top‐gate electrodes (TG). b) Cross section of the device. The high‐resistance WSe_2_ prevents the negative *V*_tg_ from being applied to the WSe_2_‐ZnO junction. The electrons in ZnO flow between the source and drain electrodes without top‐gate modulation. c,d) The device operates with light illumination. The incident light excites the photocarriers in WSe_2_, decreasing the resistance of WSe_2_ nanosheet. A conductive path for *V*_tg_ is formed between side top‐gate electrode and overlapped region. The negative *V*_tg_ can be applied to the WSe_2_‐ZnO junction. The depletion region in ZnO widens to increase the channel resistance. e) Simulated electron density distribution of the device in the dark and under 637 nm illumination. The negative *V*_tg_ (−1 V) as a reverse bias can be applied to the junction through the photoexcited WSe_2_ nanosheet, leading to an enlarged depletion region within the ZnO channel. f) Electron density along the dashed line in (e). The electron density decreases dramatically under illumination. g,h) The optical picture of the device and the corresponding spatial photocurrent map using a focused 520 nm laser with a power of 5 µW. The device was measured at *V*_ds_ = 1 V, *V*_tg_ = −0.1 V and *V*_bg_ = 0 V. Scale bar, 3 µm. i,j) Photocurrent line trace along the white dashed arrow in the inset. The blue dashed lines indicate the effective photosensitive area.

Figure 1g shows the optical picture of the LJFET. First, ZnO belts were transferred onto a p^+^‐Si/SiO_2_ (285 nm) substrate. Then, the source/drain (S/D) and side top‐gate (TG) electrodes were fabricated. Finally, a WSe_2_ nanosheet was placed on the ZnO channel and side top‐gate electrode using a micromanipulator (see method). The atomic force microscope (AFM) measurements (Figure S2, Supporting Information) reveal that the ZnO and WSe_2_ heights are ≈182 and ≈82 nm, respectively. The channel length of the LJFET is ≈20 µm. The channel width near the source (drain) electrode is ≈3 (7.5) µm. Figure 1h shows the corresponding spatially resolved photocurrent map. Photocurrent occurs on the portion of the WSe_2_ nanosheet that covers between the side top‐gate electrode and the junction, thus defining the effective photosensitive area (95.73 µm^2^, see Figure S3, Supporting Information). The intensity distribution of photocurrent perpendicular and parallel to the channel as a function of distance are plotted in Figure 1i,j, respectively. Photocurrent decreases rapidly beyond the effective photosensitive area. Light excitation will decrease the resistance of top‐gate material, thus opening a conductive path for *V*_tg_ to modulate the channel conductance. Note that there is a low photocurrent area (dark spot) near the side top‐gate electrode in the map. When the focused light scanned this area, its conductivity experienced a smaller change compared with other parts of WSe_2_ nanosheet. This may arise from its inhomogeneous photosensitive characteristics.

To further explain the gain mechanism, we compare our device with previously developed high‐gain phototransistors, including photo‐field‐effect transistor (photo‐FET) and PVFET (see **Table** **1**). For the photo‐FET based on photogating effect, the gain is proportional to the carrier lifetime. This mechanism to achieve high gain inevitably causes a *G*–*t* tradeoff. For the PVFET based on photovoltaic effect, the gain is a function of photovoltage. Strongly light‐absorbing material has to be used as the sensitizer to produce a significant photovoltage at the sensitizer‐channel junction. The gain mechanism of LJFET is distinct from the first two. The photoactive top‐gate material controls the externally applied *V*_tg_ (not photogate or photovoltage) to alter the channel conductance. The gain is determined by the modulation of *V*_tg_, and the response time is determined by the switching speed of LJFET. This architecture is effective in breaking the *G*–*t* tradeoff. Moreover, the property of strong light absorption for sensitizers is not necessary.

**Table 1:** Comparison of operating mechanisms for three types of high‐gain phototransistors

Photo‐FET	$G=\frac{\tau_{\mathrm{lifetime}}}{\tau_{\mathrm{transit}}}\;=\;\tau_{\mathrm{lifetime}}\times \;\frac{µV_{\mathrm{ds}}}{L^{2}}$	τ_lifetime_ *µ* *L* *V*_ds_	Carrier lifetime Mobility Channel length Drain–source voltage
One carrier type is captured by trapping or sensitizing centers for a certain time, while oppositely charged carrier recirculates in the conductive channel many times before recombination to generate gain which is proportional to the carrier lifetime. These devices face a tradeoff between the gain and the response time.			
PVFET	$G\;=\;\frac{N_{e}}{N_{p}}\;=\;\frac{V_{\mathrm{ph}}g_{m}/e}{P/h\nu }=V_{\mathrm{ph}}\;\times \;\frac{g_{m}h\nu }{eP}$	*V*_ph_ *g*_m_ *h* ν *e* *P*	Photovoltage Transconductance Planck's constant Photon frequency Electron charge Light power
The photovoltage that arises at the heterojunction between the photoactive material and the conductive channel generates a positive bias (like the open‐circuit voltage of a solar cell) which shrinks the depletion region, leading to an increased current in the channel. These devices need strongly light‐absorbing material as the sensitizer to produce a significant photovoltage.			
LJFET (this work)	$G=\frac{N_{e}}{N_{p}}=\frac{\Delta I_{\mathrm{ds}}/e}{P/h\nu }=\Delta I_{\mathrm{ds}}\times \frac{h\nu }{eP}$	Δ*I*_ds_ *h* ν *e* *P*	The change of *I*_ds_ induced by *V*_tg_ Planck's constant Photon frequency Electron charge Light power
The photoactive top‐gate material as a light‐driven switch controls the externally applied top‐gate voltage *V*_tg_ (not photogate or photovoltage) to alter the channel conductance (Δ*I*_ds_). The gain is determined by the modulation of *V*_tg_, and the response time is determined by the switching speed of LJFET. This device provides an efficient way to break the *G*–*t* tradeoff. Furthermore, the property of strong light absorption for sensitizers is not necessary.			

To investigate the effect of *V*_tg_ on photoresponse, transfer characteristics of LJFET under dark and light conditions with *V*_tg_ modulation were studied. It can be seen from **Figure** **2**a that there is a positive (negative) shift of threshold voltage (*V*_th_) under negative (positive) *V*_tg_ modulation even without light illumination (indicated by the black arrow). In our device, WSe_2_ nanosheet plays the role of a large resistance to weaken the *V*_tg_ effect on the ZnO channel, but it cannot totally block *V*_tg_ because it is not entirely insulated. A small fraction of negative (positive) *V*_tg_ can act on the WSe_2_‐ZnO junction in the dark, inducing a relatively wide (narrow) depletion region. Under light excitation, photocarriers are generated in WSe_2_ nanosheet. The decreased resistance of top‐gate material enables a conductive path for *V*_tg_, which modulates the channel conductance more efficiently. Because the source‐drain voltage applied to the two ends of ZnO channel was set at 1 V (source electrode is connected to the ground), the value of electrical potential at the overlapped region of ZnO channel is between 0 and 1 V. Therefore, when *V*_tg_ = 1 V (Figure 2b), the WSe_2_‐ZnO junction is forward biased. The depletion region in ZnO shrinks, leading to an increased current. A small negative shift of *V*_th_ was observed. In contrast, when *V*_tg_ = 0 V (Figure 2c), the WSe_2_‐ZnO junction is slightly reverse biased. With the increase of light power, the depth of depletion region increases. A small positive shift of *V*_th_ was detected. When *V*_tg_ = −1 V (relatively large reverse bias, Figure 2d), the depletion region becomes wider, resulting in a significant decrease in current and a large positive shift of *V*_th_.

**Figure 2:**
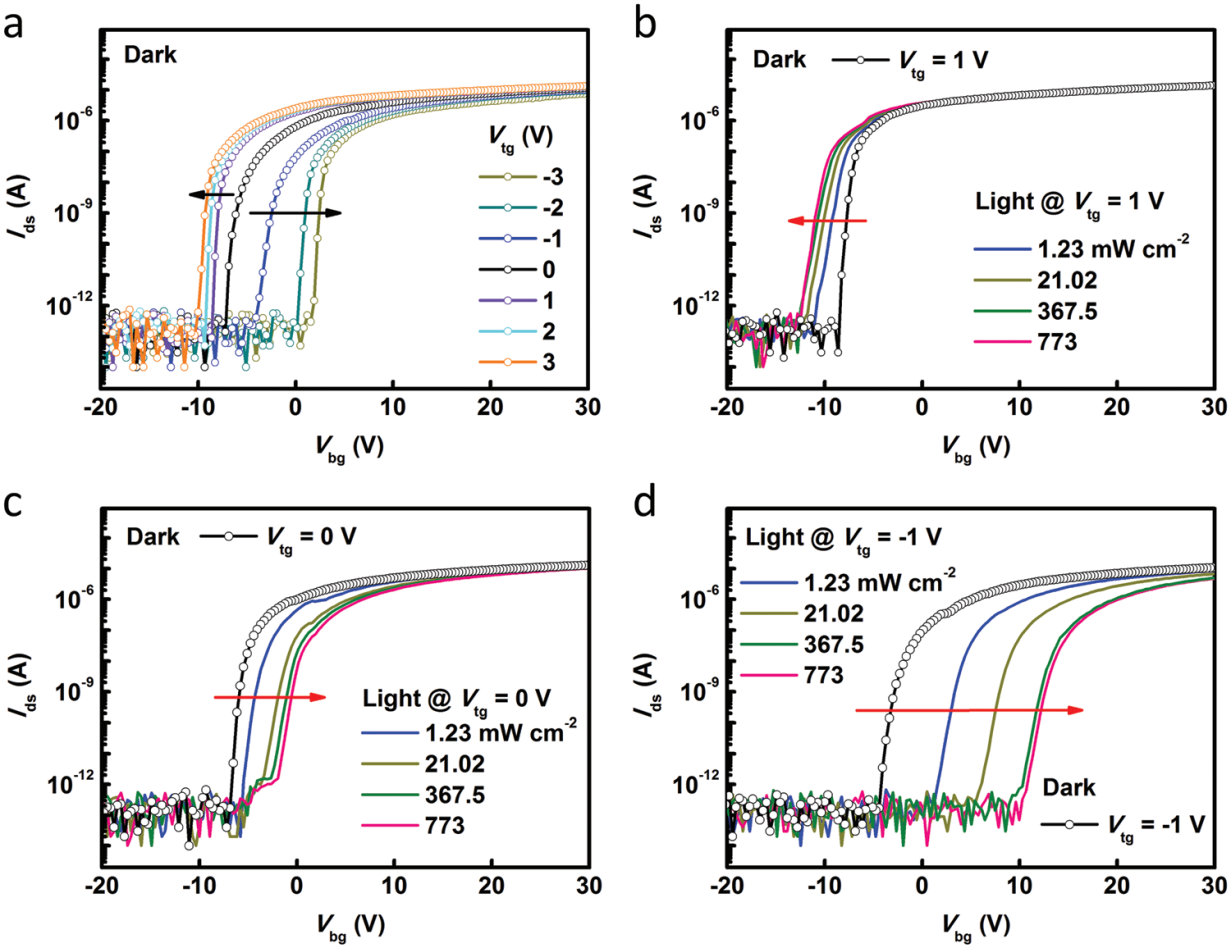
*V*_tg_ effect on transfer characteristics of WSe_2_‐ZnO LJFET under dark and light conditions. a) *I*_ds_–*V*_bg_ curves of LJFET at different *V*_tg_ of −3, −2, −1, 0, 1, 2, 3 V in the dark. b–d) *I*_ds_–*V*_bg_ curves with variable light power intensity (637 nm) at *V*_tg_ = 1, 0, and −1 V. The arrows indicate the shift direction of *V*_th_.

**Figure** **3**a shows the *I*_ds_–*V*_tg_ curves under dark and light conditions at *V*_bg_ = 15 V, and the top‐gate leakage current *I*_tg_ is given in Figure 3b. It can be seen that *I*_ds_ basically remains constant (6.6–6.1 µA) and *I*_tg_ is close to zero (10^−11^–10^−13^ A) with *V*_tg_ modulation in the dark. Upon light illumination, there is a positive net photocurrent at *V*_tg_ from 2 V to 0.6 V and a negative net photocurrent at *V*_tg_ from 0.6 to −2 V (Figure 3a). Accordingly, the polarity of *I*_tg_ flips at *V*_tg_ = 0.7 V (Figure 3b) which indicates that the electrons flow into and out of the top‐gate electrode when *V*_tg_ > 0.7 V and *V*_tg_ < 0.7 V, respectively. Here, a positive *V*_bg_ was applied to LJFET to enhance the electron density in ZnO and also keep WSe_2_ depleted of holes (high resistance), in order to achieve light‐induced rectifying junction (see Figure S4 and the relevant discussion, Supporting Information). Figure 3c–f shows the energy band diagrams of the device with illumination at different *V*_tg_. When *V*_tg_ > 0.7 V, the junction is forward biased (Figure 3c). The electrons from ZnO flow into WSe_2_, contributing to the positive *I*_tg_. Meanwhile, the depletion region in ZnO shrinks, resulting in a high *I*_ds_. When *V*_tg_ = 0.7 V, the external applied voltage difference across the junction is equal to the photovoltage (Figure 3d). No electrons pass through the junction, resulting in a zero *I*_tg_. At the same time, due to that the photovoltaic effect induces a relatively narrow depletion region in ZnO, *I*_ds_ is somewhat higher than that measured in the dark. With *V*_tg_ decreases to 0.6 V, the Fermi level of ZnO is equal to that of WSe_2_ (Figure 3e). The band alignment returns to the original state (Figure S5, Supporting Information). So, *I*_ds_ is equal to that measured in the dark. Additionally, the photoexcited electrons in WSe_2_ flow into ZnO, contributing to the negative *I*_tg_. When *V*_tg_ < 0.6 V, the junction is reverse biased (Figure 3f). The depletion region in ZnO becomes wider to pinch off the channel, resulting in a low *I*_ds_. The electrons photoexcited in WSe_2_ flow into ZnO, contributing to the negative *I*_tg_. In our device, *I*_tg_ is not more than 2 nA with illumination. The light can only change a small portion of the resistance of WSe_2_. Because the magnitude and polarity of photocurrent are tunable via field‐effect modulation, a very high responsivity can be achieved with appropriate *V*_tg_ and *V*_bg_.

**Figure 3:**
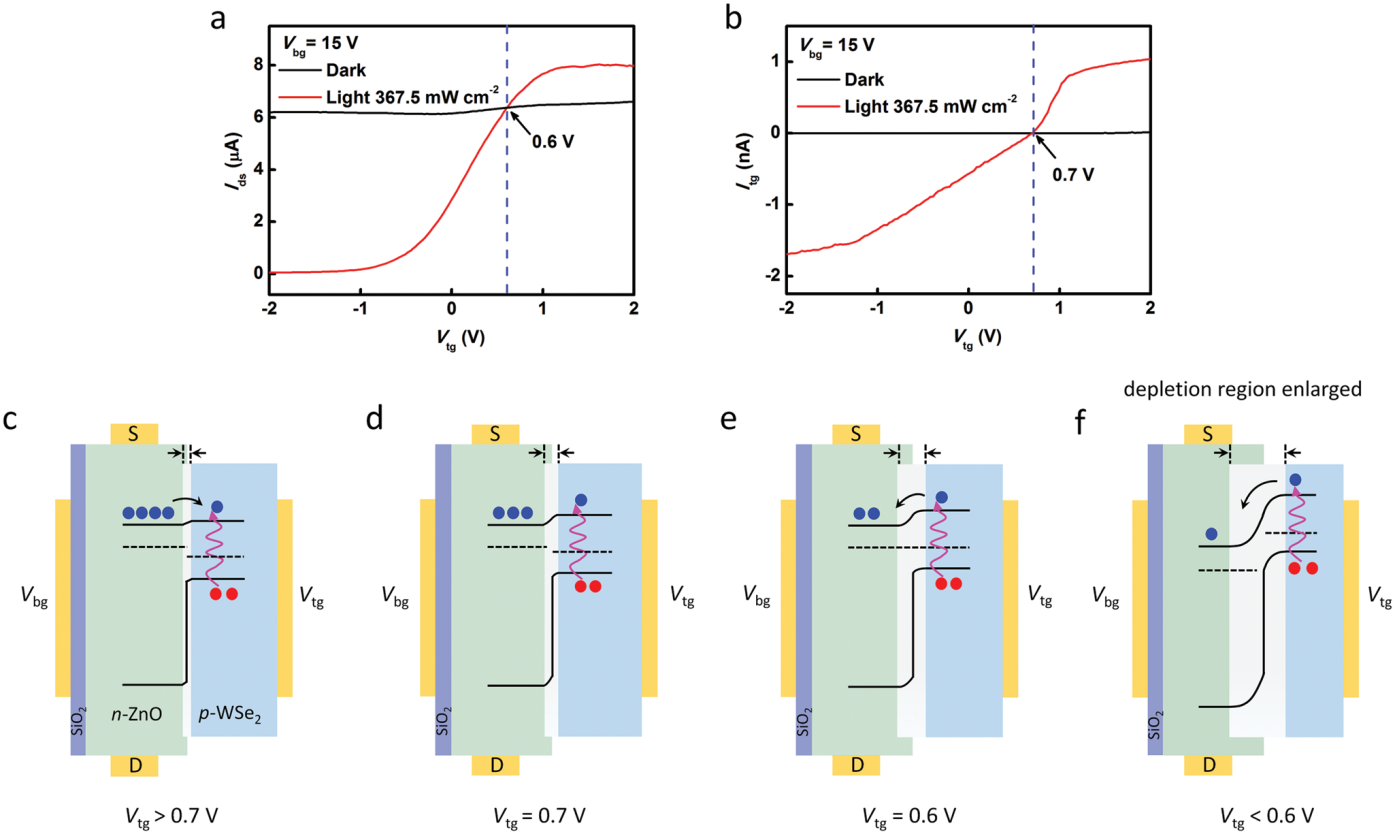
*I*_ds_–*V*_tg_ and *I*_tg_–*V*_tg_ characteristics. a) *I*_ds_–*V*_tg_ curves under dark and light conditions. There is a positive net photocurrent at *V*_tg_ from 2 to 0.6 V and a negative net photocurrent at *V*_tg_ from 0.6 to −2 V. b) *I*_tg_–*V*_tg_ curves under dark and light conditions. The polarity of *I*_tg_ flips at *V*_tg_ = 0.7 V. c–f) The energy band diagrams at the junction with illumination at different *V*_tg_. The blue and red dots represent the electrons and holes, respectively.

The *I*_ds_–*V*_ds_ curves with variable light power intensity at *V*_tg_ = −1 V and *V*_bg_ = 15 V have been shown in **Figure** **4**a. The illumination causes a distinct decrease in *I*_ds_ as marked by the dotted arrow, and a net photocurrent as high as 6.6 µA is obtained at *V*_ds_ = 1 V and 367.5 mW cm^−2^. Accordingly, Figure S6 (Supporting Information) shows the *I*_ds_–*V*_bg_ and *I*_ds_–*V*_ds_ curves of a bare WSe_2_ transistor by applying the voltage to the two side top‐gate electrodes underneath the WSe_2_ nanosheet (see Figure 1g). With a power intensity of 367.5 mW cm^−2^, the anomalous photocurrent reaches a maximum of 6.6 µA, and the WSe_2_ transistor only shows a photocurrent about 2 nA at *V*_ds_ = 1 V. Although the illumination induces a small increase of the photocurrent in WSe_2_, the small change in the resistance of WSe_2_ arouses a remarkable field‐effect modulation in LJFET. Figure S7 (Supporting Information) shows the simulated electron density distribution of LJFET with illumination. The electron density of ZnO channel decreases with an increase of power intensity. Figure S8 (Supporting Information) presents the photoresponse properties of WSe_2_‐ZnO PVFET. A positive photocurrent of ≈0.25 µA can be obtained. In this PVFET, photovoltage is not large enough to generate a high photocurrent compared to that in LJFET. This supports the simulation results.

**Figure 4:**
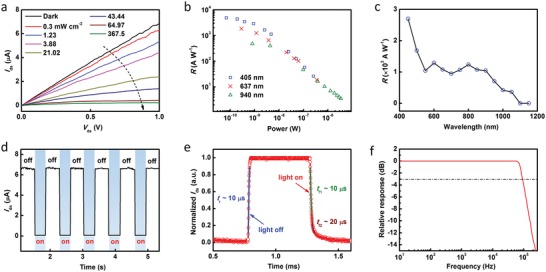
Photoresponse performance. a) *I*_ds_–*V*_ds_ curves with variable light power intensity (637 nm) at *V*_tg_ = −1 V and *V*_bg_ = 15 V. b) Responsivities versus light power for 405, 637, and 940 nm illumination. c) Spectral responsivity of WSe_2_‐ZnO LJFET. The power intensity remained constant at 1 mW cm^−2^ for different wavelengths. To avoid the response of ZnO, the wavelength is varied from 450 to 1150 nm with an interval of 50 nm. d) Temporal response of the device for 637 nm illumination at *V*_tg_ = −1 V and *V*_bg_ = 15 V. The power intensity is 367.5 mW cm^−2^. e) A single modulation cycle. The rise time is ≈10 µs. The fall time consists of a fast component of ≈10 µs and a slow component of ≈20 µs. f) Relative photoresponse as a function of light modulation frequency.

In our device, ZnO with a bandgap of ≈3.3 eV is sensitive only to ultraviolet radiation (Figure S9, Supporting Information). Therefore, the WSe_2_ nanosheet with a bandgap of ≈1.2 eV indicates a broadband photoresponse from the visible to near‐infrared range. Figure S10 (Supporting Information) presents the *I*_ds_–*V*_ds_ curves with 405 and 940 nm light illumination. Both illumination wavelengths caused a distinct decrease in *I*_ds_. The responsivities as a function of the light power for different wavelengths are summarized in Figure 4b. The responsivities as high as 4.83 × 10^3^, 1.84 × 10^3^ and 4.82 × 10^2^ A W^−1^ are obtained for illumination with 405, 637, and 940 nm light, respectively. The higher responsivity with higher photon energy can be attributed to the more efficient excitation of photocarriers in WSe_2_. Moreover, the spectral responsivity of the device has been shown in Figure 4c. In the visible region, the responsivity is as high as ≈10^3^ A W^−1^. As the wavelength increases to near infrared, the responsivity decreases to ≈10^2^ A W^−1^. Clearly, the cut‐off wavelength is determined by the bandgap of WSe_2_ nanosheet. The gain for this LJFET is defined as the number of charges collected by the source‐drain electrodes due to the excitation by one photon and given by *G* = Δ*I*_ds_ × *hν*/*eP* 38 (parameters have been defined in Table 1). Assuming that light incident on the effective photosensitive area is absorbed completely, a gain as high as 1.48 × 10^4^ is obtained.

Response time is a key parameter that determines the capability of a photodetector to follow a fast‐varying optical signal. As shown in Figure 4d, the anomalous photoresponse exhibits highly stable and reproducible characteristics with an *I*_off_/*I*_on_ ratio of 100 under light modulation. Figure 4e gives the temporal response in a single modulation cycle. The rise edge, defined as the time necessary for a current increase from 10% *I*_peak_ to 90% *I*_peak_ is as fast as 10 µs, indicating a rapid carrier recombination in WSe_2_. The fall edge consists of a fast component for a current decrease from 90% *I*_peak_ to 35% *I*_peak_ (≈10 µs) followed by a slow component from 35% *I*_peak_ to 10% *I*_peak_ (≈20 µs). The former indicates the fast generation of photocarriers in WSe_2_, and the latter suggests that ZnO channel undergoes a slightly slow process to reach steady state under *V*_tg_ modulation. Figure S11 (Supporting Information) presents the temporal response with 405 and 940 nm illumination and shows sharp rise and fall edges, proving that this LJFET device, together with the measurement in Figure 4d, possesses a fast and broadband photoresponse.

In order to study the working bandwidth of WSe_2_‐ZnO LJFET, a relative a.c. photoresponse measurement was performed using a modulated optical signal over a broad frequency range from 10 Hz to 250 kHz. It can be seen from Figure 4f that the device presents a broadband frequency response with a 3 dB bandwidth of 90 kHz. A gain‐bandwidth product as high as 1.3 × 10^9^ Hz was obtained. This value is comparable to that of reported high‐gain phototransistors.^18^, ^31^ In our work, the high gain‐bandwidth product is achieved by decoupling the gain from the carrier lifetime and without the assistance from strongly light‐absorbing materials. This value can be further improved by decreasing the defects in photosensitive material, channel material and interface, which are considered as the main factors affecting response time and bandwidth.^31^ In addition, the noise current measurement of LJFET was conducted and it was found that flicker noise (1/*f* noise) dominates at low frequencies and white noise dominates at high frequencies (Figure S12, Supporting Information). According to the values of noise current and responsivity, a detectivity of 1.56 × 10^13^ cm Hz^1/2^ W^−1^ for 405 nm illumination was obtained. Figure S13 (Supporting Information) gives the temporal response of the other three WSe_2_‐ZnO LJFETs with relatively small dimensions. These devices show fast photoresponse but a little decrease in photocurrent maybe due to the narrow channel width. In our design, although the size and quality of channel materials have impact on the photoresponse of LJFET, the photocurrent can be enhanced by modulating *V*_bg_ and *V*_tg_.

To demonstrate that our design is a general approach for high‐performance photodetectors, a different gate material, GaSe, was employed to pair with ZnO (**Figure** **5**a). According to the AFM profiles (Figure S14, Supporting Information), the heights of ZnO and GaSe (bandgap of ≈2.0 eV,^39^ corresponding to 620 nm light) are ≈53 and ≈28 nm, respectively. Figure 5b presents the *I*_ds_–*V*_bg_ curves under dark and light conditions. Similar to the WSe_2_‐ZnO LJFET, an obvious shift of the *V*_th_ is observed with illumination. With the *V*_tg_ modulation, positive and negative net photocurrent can also be obtained (Figure 5c). Due to the large resistance of GaSe, only a small fraction of *V*_tg_ can be applied to the junction even with illumination. So, the voltage at the crossing point in Figure 5c is greater than the source‐drain bias of 1 V. A net photocurrent of 2.74 µA is obtained at *V*_ds_ = 1 V and 0.414 mW cm^−2^ (Figure 5d). Figure S15 (Supporting Information) shows the *I*_ds_–*V*_ds_ and *I*_ds_–*V*_bg_ curves of a bare GaSe transistor by applying the voltage to the two side top‐gate electrodes underneath the GaSe nanosheet. When the anomalous photocurrent of LJFET reaches its maximum with a power intensity of 0.414 mW cm^−2^, the photocurrent of the bare GaSe transistor is lower than 30 pA. Interestingly, the GaSe itself does not show good photoresponse, but the GaSe photoexcited in our architecture displays a significant field‐effect modulation. Responsivity as high as 1.7 × 10^4^ A W^−1^ is obtained (Figure 5e). This result is three orders of magnitude superior to the previously reported responsivity of GaSe based photodetectors.^39^, ^40^ Figure 5f gives a single modulation cycle of temporal response. The rise and fall edges are 165 and 144 µs, respectively, which is slower than that of WSe_2_‐ZnO LJFET. In order to study the effect of gate material on response speed of LJFET, Figure S16 (Supporting Information) gives the measurements of temporal response for other GaSe‐ZnO LJFETs and Table S1 (Supporting Information) summarizes the response time of reported typical bare GaSe and WSe_2_ photodetectors. The results show that the quality and type of photosensitive material have a significant effect on the device performance. The fact that response speed of WSe_2_‐ZnO LJFET is faster than that of GaSe‐ZnO LJFET may be attributable to the difference in photoelectric conversion efficiency and defect states between different gate materials.

**Figure 5:**
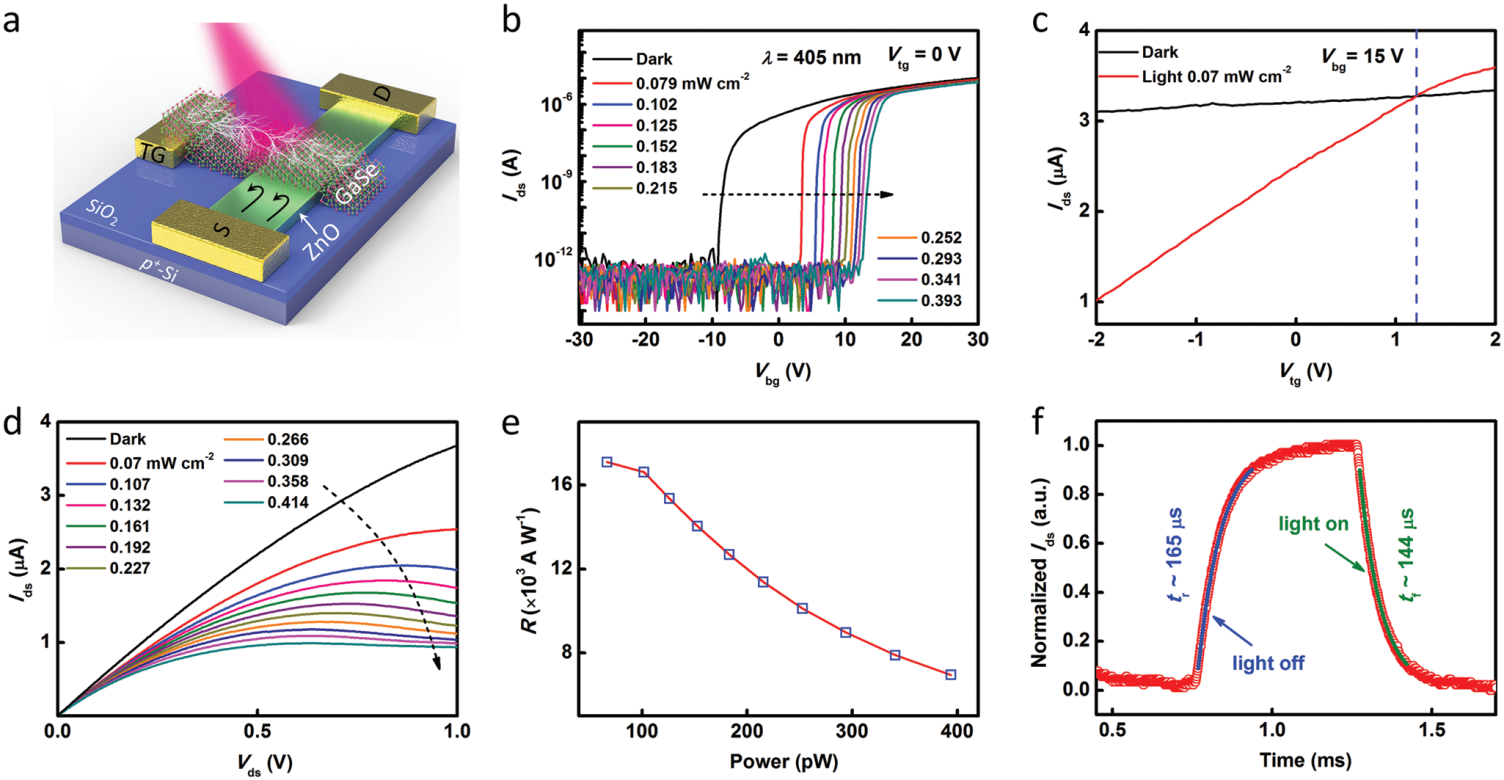
Photoresponse of GaSe‐ZnO LJFET. a) A schematic illustration of the device with illumination. The photocarriers in GaSe excited by light illumination induce a conductive path for *V*_tg_. b) *I*_ds_–*V*_bg_ curves with variable light power intensity (405 nm) at *V*_tg_ = 0 V. c) *I*_ds_–*V*_tg_ curves under dark and light conditions. The polarity of photocurrent flips at *V*_tg_ = 1.2 V. d) *I*_ds_–*V*_ds_ curves with variable power intensity (405 nm) at *V*_tg_ = 0 V and *V*_bg_ = 15 V. e) Responsivity versus light power for 405 nm illumination. The effective photosensitive area is 95.15 µm^2^ (see Figure S14, Supporting Information). f) A single modulation cycle. The rise and fall times are ≈165 and ≈144 µs, respectively.

In summary, we have developed a novel LJFET for high‐efficiency photodetection. In this architecture, the p‐type material plays a role of a light‐driven switch, controlling the externally applied top‐gate voltage to alter the n‐type channel conductance. As a result, the gain is determined by the field‐effect modulation, and the response time is determined by the switching speed of LJFET. Different from trap‐assisted high‐gain phototransistors, our devices possess high responsivity and fast response time over a broad spectrum through decoupling the gain from carrier lifetime. The performance can be further improved by appropriate choices of ideal gate materials and transport channels. The simple architecture provides an efficient and reliable way to break the *G*–*t* tradeoff for high‐gain photodetectors.

## Experimental Section

*ZnO Growth*: ZnO belts used in this work were prepared by a chemical vapor transport method. Equal amounts of ZnO (99.99%, purity) and graphite (99.9%, purity) powders mixed with a 2.5% weight percentage of P_2_O_5_ (99.99%, purity) powder as precursors were loaded into a quartz boat that was placed in the center of a tube furnace. A Si/SiO_2_ (285 nm) substrate coated with 1 nm thick Au catalyst film was placed on the boat. High‐purity Ar (70 sccm, flow rate) and O_2_ (15 sccm, flow rate) were used as the carrier gases. The furnace was heated to 1000 °C within 30 min and maintained at that temperature for 10 min. Finally, the samples were cooled to room temperature naturally.

*Device Fabrication*: First, ZnO belts were transferred mechanically onto p^+^‐Si/SiO_2_ (285 nm) substrate by direct contact with the sample substrate. The source, drain, and side top‐gate electrodes (Cr/Au) were prepared by electron‐beam lithography, metallization, and the lift‐off process. Then, the WSe_2_ or GaSe nanosheet was mechanically exfoliated on a poly‐dimethyl siloxane (PDMS) layer. A micromanipulator was used to put the WSe_2_ or GaSe nanosheet, which is adhered to PDMS, onto the target ZnO channel and the side top‐gate electrodes to form the LJFET structure using the microscope to locate the position. Finally, the WSe_2_ or GaSe nanosheet was released from PDMS through heating the substrate.

*Photoresponse Characterization*: Photoresponse measurements of LJFET were conducted using a Lake Shore Probe Station with an Agilent B1500 semiconductor parameter analyzer and laser diodes with the wavelengths of 405, 637, and 940 nm. For the temporal photoresponse measurements, an oscilloscope was used to monitor the time dependence of the current. For the scanning photocurrent microscopy measurements, a galvanometer mirror scanning system was used to achieve the laser spot scanning across the device. The device is mounted onto a sample stage, and then illuminated with a focused 520 nm laser using a × 100/NA‐0.9 objective. The laser was modulated at a frequency of ≈817 Hz with a controller as the reference to a lock‐in amplifier from which the photocurrent signal could be extracted. Spectral response measurement was performed using a supercontinuum spectrum laser source combined with a monochromator and Agilent B2902. For the bandwidth measurement, an arbitrary waveform generator was used to drive 405 nm laser, achieving high‐frequency light modulation, and a lock‐in amplifier was used to acquire photocurrent.

*Device Simulations*: Device simulations were performed using Sentaurus TCAD. The model used in the software was based on drift‐diffusion method, where the Poisson's equation and continuity equations for electrons and holes were solved self‐consistently by the finite element approach. In order to present the electron density distribution in the cross section of the device more clearly, the device thickness was increased and the channel length was shortened during the simulation. The doping concentrations of p‐region and n‐region were 10^11^ and 10^15^ cm^−3^, respectively.

## Conflict of Interest

The authors declare no conflict of interest.

## Supporting information

Supporting Information
