# Supplementary material for: Light‐Driven WSe2‐ZnO Junction Field‐Effect Transistors for High‐Performance Photodetection
Source: Adv Sci (Weinh). 2019 Nov 11;7(1):1901637. doi: 10.1002/advs.201901637 (PMC6947501; doi:10.1002/advs.201901637)
Supplement: Supplementary file 1 — Supporting Information [file ADVS-7-1901637-s001.pdf]

Copyright WILEY-VCH Verlag GmbH & Co. KGaA, 69469 Weinheim, Germany, 2019.

ADVANCED SCIENCE
Open Access

Supporting Information

for *Adv. Sci.,* DOI: 10.1002/advs.201901637

Light-Driven WSe_2-ZnO Junction Field-Effect Transistors for High-Performance Photodetection

Nan Guo, Lin Xiao, Fan Gong, Man Luo, Fang Wang, Yi Jia, Huicong Chang, Junku Liu, Qing Li, Yang Wu, Yang Wang, Chongxin Shan, Yang Xu, Peng Zhou,* and Weida Hu**

WILEY-VCH

Copyright WILEY-VCH Verlag GmbH & Co. KGaA, 69469 Weinheim, Germany, 2019.

Supporting Information

Light-Driven WSe_2-ZnO Junction Field-Effect Transistors for High-Performance Photodetection

Nan Guo, Lin Xiao, Fan Gong, Man Luo, Fang Wang, Yi Jia, Huicong Chang, Junku Liu, Qing Li, Yang Wu, Yang Wang, Chongxin Shan, Yang Xu, Peng Zhou*, and Weida Hu**

Contents

S1: Structure and operating mechanism of WSe_2-ZnO PVFET

S2: AFM measurements of WSe_2-ZnO LJFET

S3: Calculation of photosensitive area of WSe_2-ZnO LJFET

S4: Light-induced rectifying junction

S5: Band diagram of the WSe_2-ZnO junction

S6: Power dependence of I_{ds}-V_{bg} and I_{ds}-V_{ds} characteristics of bare WSe_2 transistor

S7: Simulated electron density distribution of the device with different power intensities

S8: Photoresponse of WSe_2-ZnO PVFET

S9: I_{ds}-V_{bg} characteristics of bare ZnO transistor with different illumination wavelengths

S10: Power dependence of I_{ds}-V_{ds} curves of WSe_2-ZnO LJFET with 405 and 940 nm illumination

S11: Temporal response of WSe_2-ZnO LJFET with 405 and 940 nm illumination

S12: Noise current measurement

S13: Temporal response of WSe_2-ZnO LJFETs with relatively small dimensions

S14: AFM measurements of GaSe-ZnO LJFET

S15: I_{ds}-V_{bg} and I_{ds}-V_{ds} characteristics of bare GaSe transistor

S16: Response time of other GaSe-ZnO LJFETs

References

S1: Structure and operating mechanism of WSe_2-ZnO PVFET

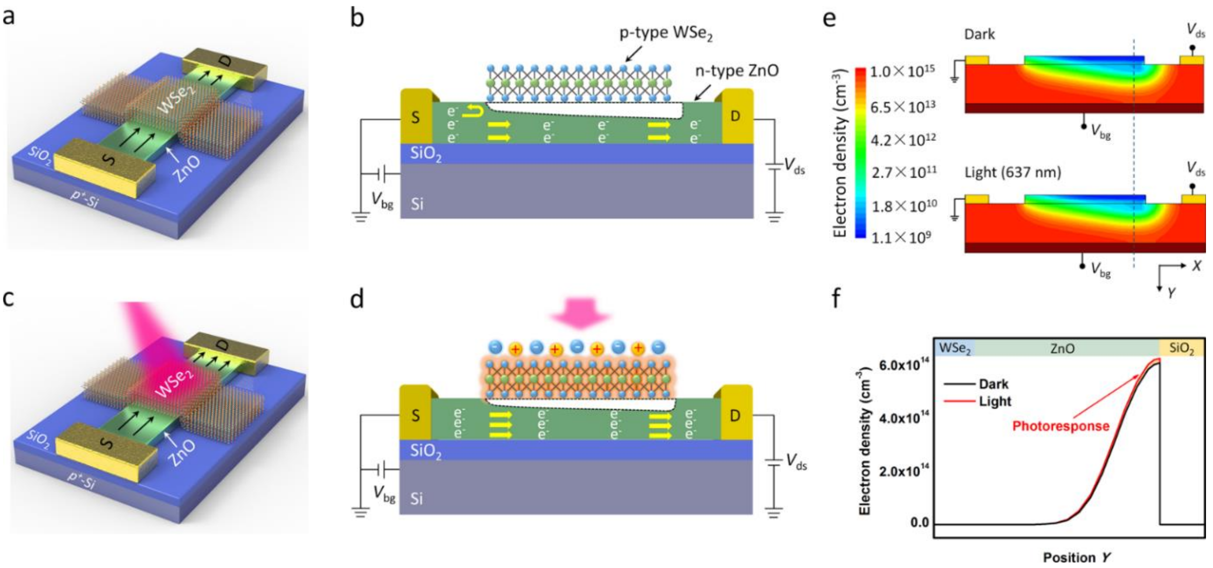

Figure S1. WSe_2-ZnO PVFET. (**a,b**) A schematic illustration of the device in the dark. The p-WSe_2 nanosheet as the photoactive material is transferred onto the n-ZnO channel. The depletion region is formed at WSe_2-ZnO junction. (**c,d**) The device operates with light illumination. The incident light excites the photocarriers in WSe_2, producing a photovoltage. This photovoltage as a positive bias shrinks the depletion region within the ZnO channel, leading to an increased I_{ds}. (**e,f**) Simulated electron density distribution of the device in the dark and under 637 nm illumination. The photovoltage generated at the junction results in a small increase in electron density.

S2: AFM measurements of WSe_2-ZnO LJFET

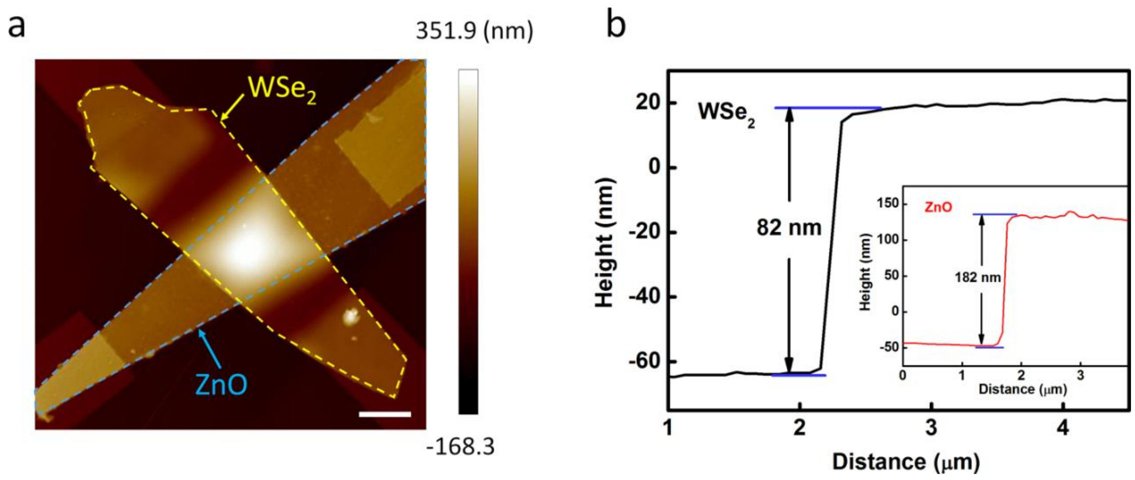

Figure S2. (**a**) AFM image of the WSe_2-ZnO LJFET. Scale bar, 3 μm. (**b**) Height profiles of the WSe_2 nanosheet (black line) and the ZnO belt (red line).

S3: Calculation of photosensitive area of WSe_2-ZnO LJFET

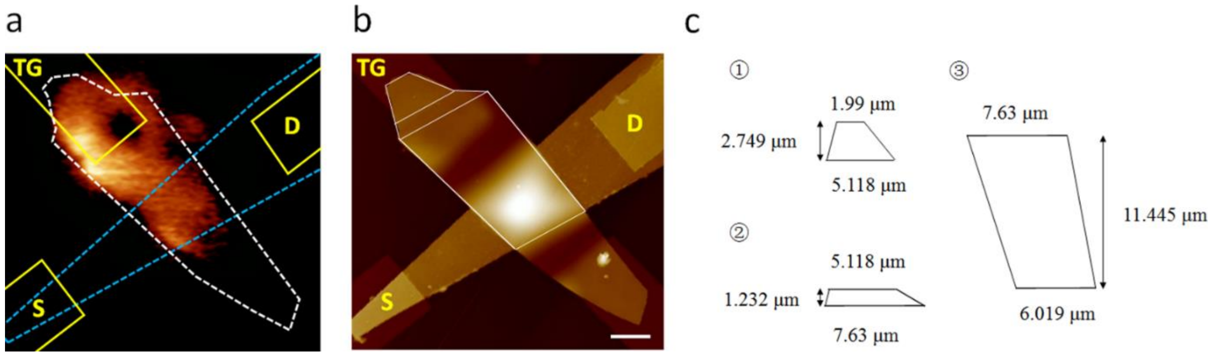

Figure S3. (**a**) Scanning photocurrent map of WSe_2-ZnO LJFET (520 nm laser, 5 μW) reveals the effective photosensitive area between the side top-gate electrode and the overlapped region. (**b**) AFM image of WSe_2-ZnO LJFET. Scale bar, 3 μm. The effective photosensitive area indicated by the white line was divided into three trapezoids. (**c**) Size of trapezoids used for calculation of effective photosensitive area (95.73 μm^2).

S4: Light-induced rectifying junction

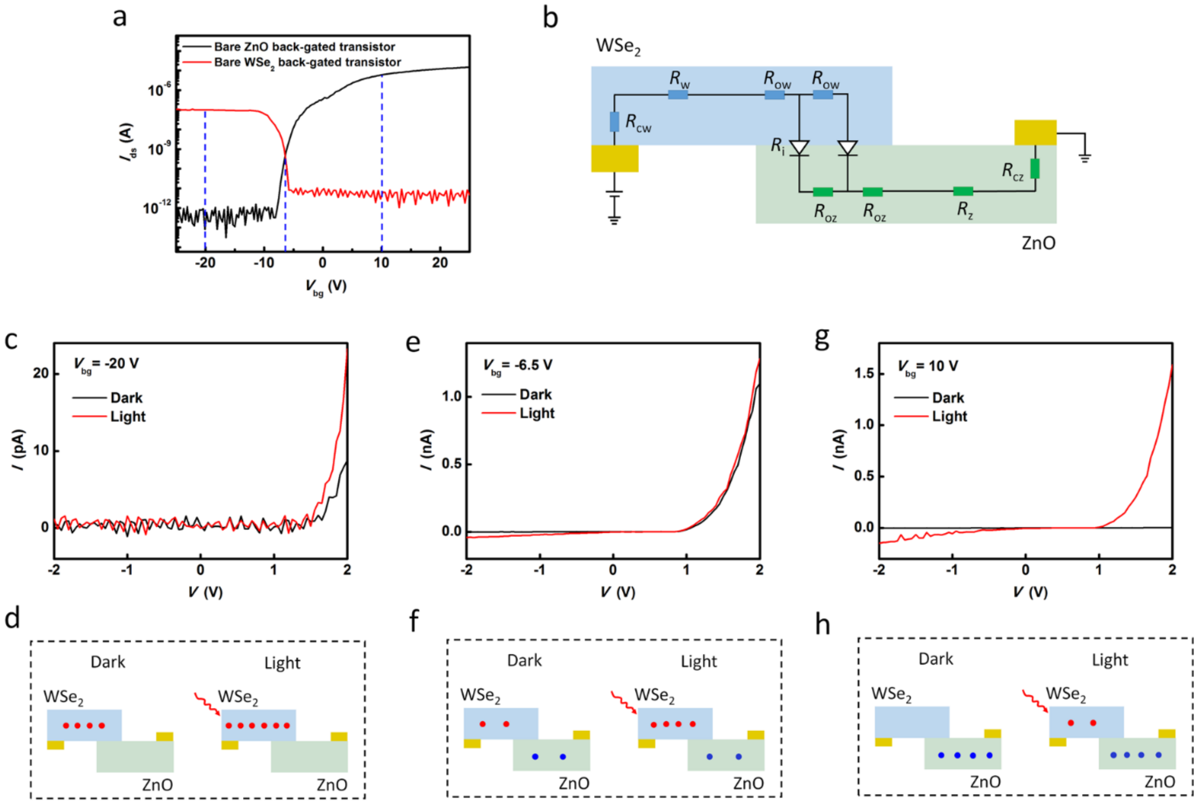

Figure S4. (**a**) I_{ds}-V_{bg} curves of WSe_2 (red line) and ZnO (black line) back-gated transistors. The I_{ds}-V_{bg} measurement of WSe_2 back-gated transistor is conducted by applying the source-drain voltage to the two side top-gate electrodes underneath the WSe_2 nanosheet (see Figure 1g). (**b**) A schematic of the WSe_2-ZnO heterostructure device model, where R_{cw} (R_{cz}) is the contact resistance between WSe_2 (ZnO) and the electrode; R_w (R_z) represents the WSe_2 (ZnO) series resistance outside the overlapped region; R_{ow} (R_{oz}) is the WSe_2 (ZnO) resistance in the overlapped region; and R_i represents the junction resistance. (**c-h**) Back-gate tunable *I-V* characteristics of the WSe_2-ZnO heterostructure device. The power intensity is 367.5 mW cm^{-2} (637 nm).

In order to study the rectifying characteristics of the WSe_2-ZnO heterostructure with V_{bg} modulation, we perform the I_{ds}-V_{bg} measurements of WSe_2 and ZnO back-gated transistors first. From Figure S4a, it can be seen that when $V_{bg} < -7.8$ V, the carriers in ZnO (WSe_2) are depleted (enhanced); when $-7.8 \text{ V} < V_{bg} < -5.8$ V, there are some carriers in both ZnO and WSe_2; when $V_{bg} > -5.8$ V, the carriers in ZnO (WSe_2) are enhanced (depleted). So, the applied

V_{bg} can cause one material to remain conductive and the other to remain non-conductive except for the V_{bg} range between -7.8 V and -5.8 V. Figure S4b depicts a simplified device model for the WSe_2-ZnO heterostructure. Figure S4c shows the *I*-*V* curves of the device at V_{bg} = -20 V (the voltage was marked by the blue dashed line in Figure S4a). The currents are very low even under light illumination. The device does not show a good rectifying characteristic. This is because that the V_{bg} of -20 V depleted the carriers in ZnO and enhanced the carriers in WSe_2 (Figure S4d). ZnO functions as a large resistance for the heterostructure. The illumination can only change the resistance of WSe_2 but not that of ZnO, resulting in low currents under both dark and light conditions. Figure S4e shows the *I*-*V* curves of the device at V_{bg} = -6.5 V. The device shows rectifying characteristics under dark and light conditions. This is because that both ZnO and WSe_2 remain conductive at V_{bg} = -6.5 V (see Figure S4a and Figure S4f). The illumination can further decrease the resistance of WSe_2. So, the device presents a rectifying state. Figure S4g shows the *I*-*V* curves of the device at V_{bg} = 10 V. The device shows a rectifying characteristic only under light excitation. This is because the V_{bg} of 10 V depleted the carriers in WSe_2 and enhanced the carriers in ZnO (see Figure S4a and Figure S4h). In the dark, WSe_2 functions as a large resistance for the heterostructure, resulting in a low dark current. Upon light illumination, the carriers are excited in WSe_2 (see Figure S4h) and both ZnO and WSe_2 remain conductive. The rectifying state of the heterostructure is induced by light. This is our design basis of LJFET.

S5: Band diagram of the WSe_2-ZnO junction

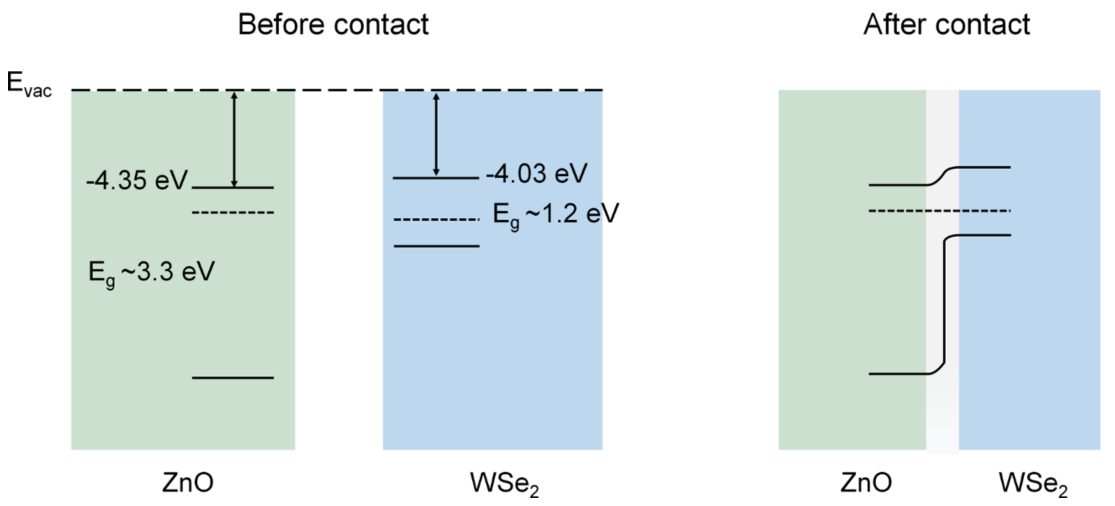

Figure S5. Energy band diagrams of the WSe_2-ZnO junction at V_{bg} = 15 V without V_{ds} and V_{tg} modulation. According to the transfer characteristics shown in Figure S4a, when V_{bg} = 15 V, the Fermi level of n-type ZnO is near the conduction band edge and that of p-type WSe_2 is close to the middle of bandgap. The electron affinity of ZnO and WSe_2 are -4.35 eV and -4.03 eV, and the bandgap of ZnO and WSe_2 are ~3.3 eV and ~1.2 eV[1,2], respectively.

S6: Power dependence of I_{ds}-V_{bg} and I_{ds}-V_{ds} characteristics of bare WSe_2 transistor

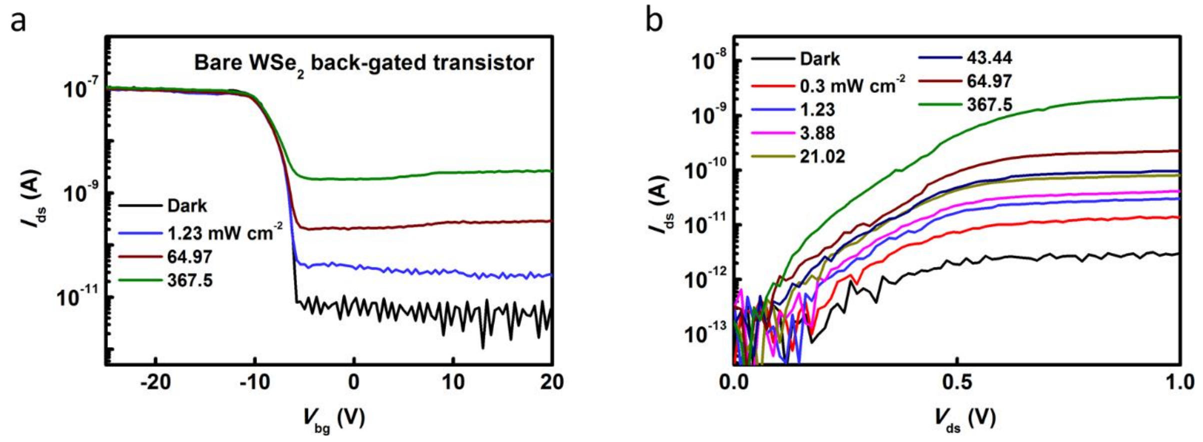

Figure S6. I_{ds}-V_{bg} (**a**) and I_{ds}-V_{ds} (**b**) curves of bare WSe_2 transistor with variable light power intensity (637 nm). The source-drain voltage is applied to the two side top-gate electrodes which are underneath the WSe_2 nanosheet (see Figure 1g). In Figure b, V_{bg} of 15 V was used.

S7: Simulated electron density distribution of the device with different power intensities

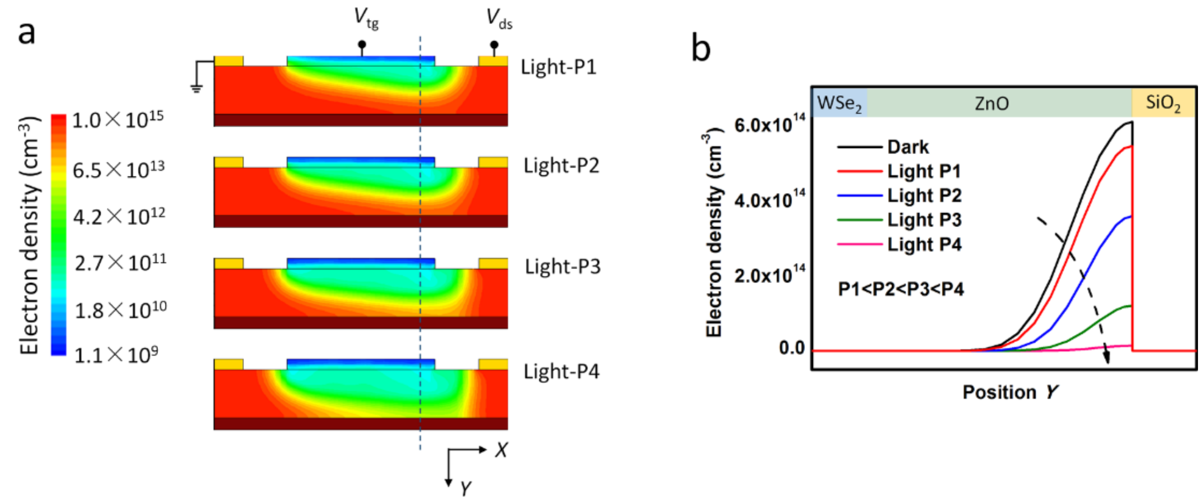

Figure S7. Simulated electron density distribution of the device in the dark and under 637 nm illumination with different power intensities.

S8: Photoresponse of WSe_2-ZnO PVFET

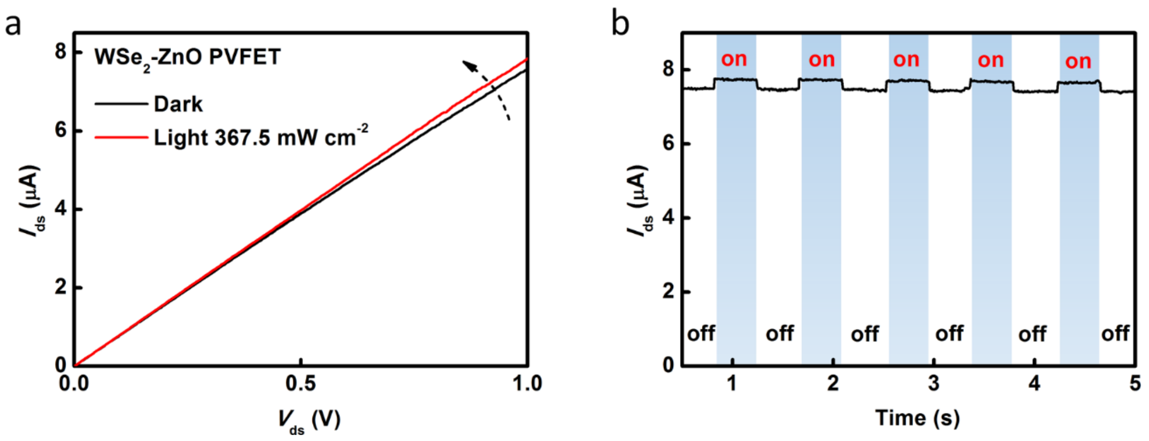

Figure S8. I_{ds}-V_{ds} curves (**a**) and temporal response (**b**) of WSe_2-ZnO PVFET for 637 nm illumination. A small positive photocurrent induced by photovoltage can be observed.

S9: I_{ds}-V_{bg} characteristics of bare ZnO transistor with different illumination wavelengths

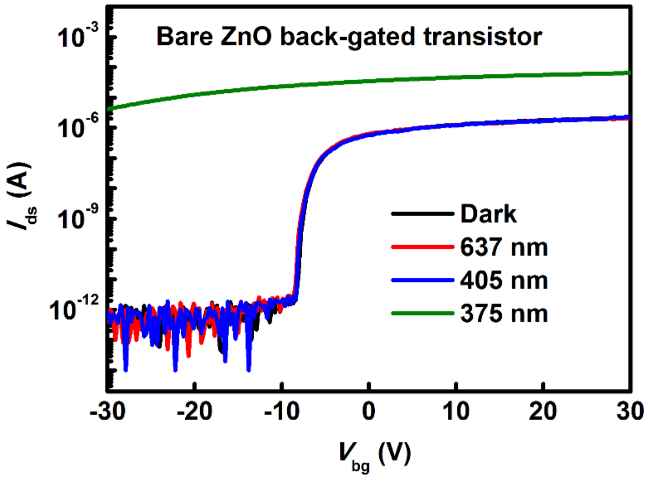

Figure S9. I_{ds}-V_{bg} characteristics of bare ZnO transistor with different illumination wavelengths of 375, 405 and 637 nm. Power intensity is 1 mW cm^{-2}. The photocurrent is remarkable only under 375 nm light illumination due to the ~3.3 eV bandgap of ZnO. Therefore, in our device, only WSe_2 is responsible for the photoresponse when the photon energy is below the bandgap of ZnO.

S10: Power dependence of I_{ds}-V_{ds} curves of WSe_2-ZnO LJFET with 405 and 940 nm illumination

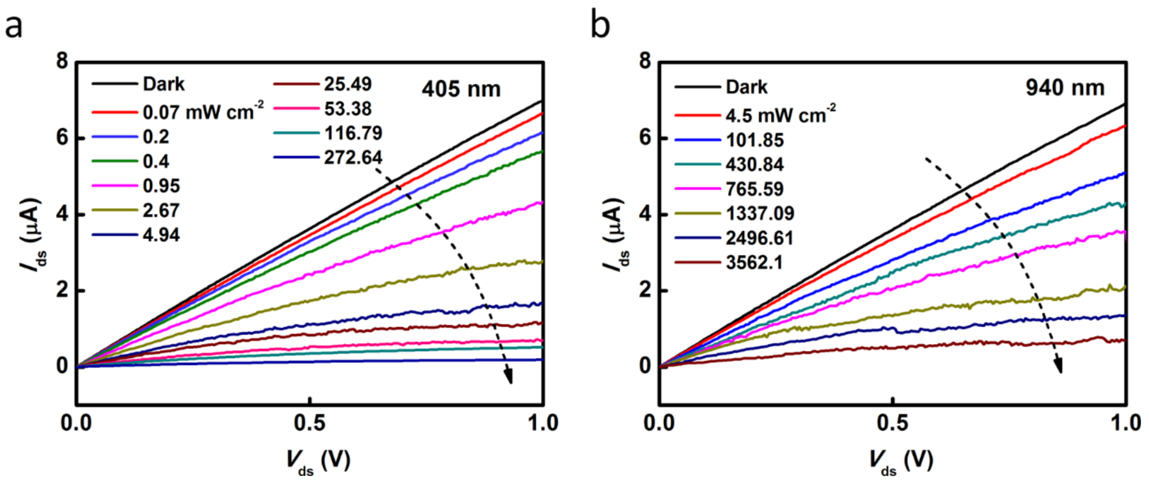

Figure S10. I_{ds}-V_{ds} curves with variable light power intensity (405 and 940 nm) at V_{tg} = -1 V and V_{bg} = 15 V. It can be seen that there is a distinct decrease in I_{ds} (marked by the dashed arrow), which is the same in the measurement with 637 nm light illumination. Moreover, the higher photon energy can excite the photocarriers in WSe_2 more efficiently at lower excitation power.

S11: Temporal response of WSe_2-ZnO LJFET with 405 and 940 nm illumination

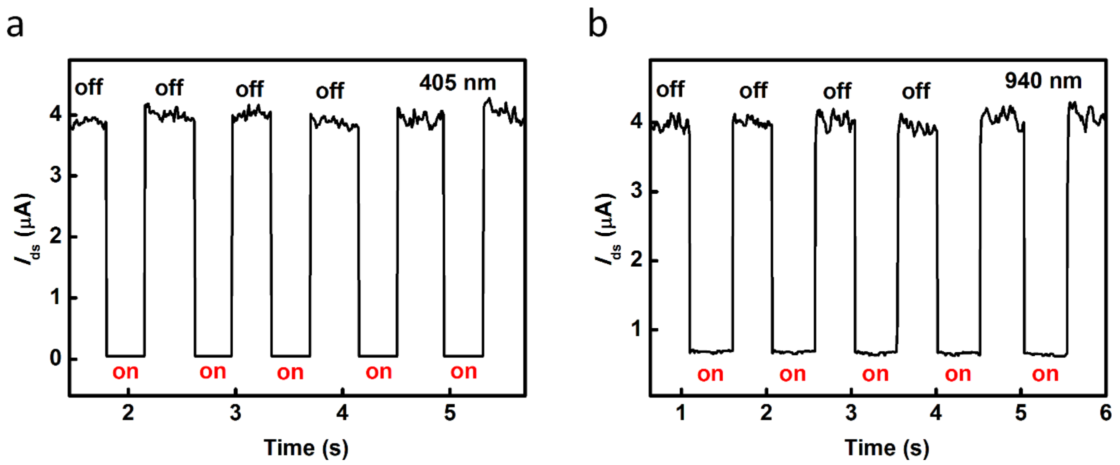

Figure S11. Temporal response of another device in our work for 405 nm (**a**) and 940 nm (**b**) illumination.

S12: Noise current measurement

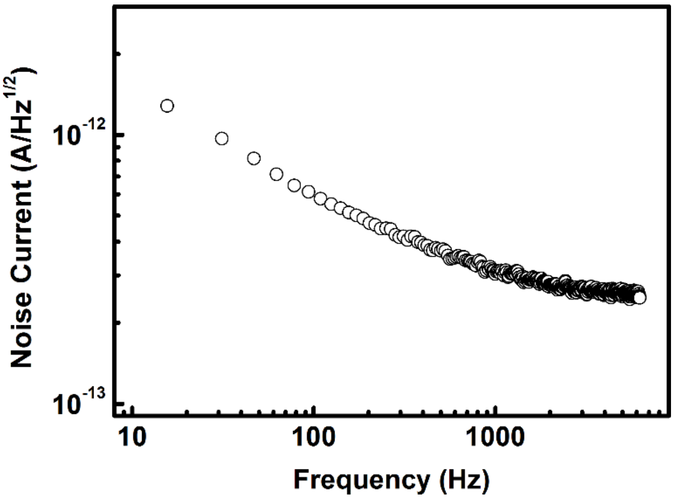

Figure S12. Noise current measurement of WSe_2-ZnO LJFET.

Noise current measurement was conducted using a low-noise current preamplifier and a FFT spectrum analyzer. The device was kept in a metal box which provides a shielded and dark environment. The source-drain current is ~6.5 μA. It can be seen from Figure S12 that flicker noise (1/*f* noise) dominates at low frequencies and white noise dominates at high frequencies. According to the frequency dependence results, we calculate the specific detectivity (D^*) at f = 1 kHz. D^* is defined as $D^* = (A\Delta f)^{1/2} R / i_n$, where A is the effective area of the device, R is the responsivity, Δf is the electrical bandwidth, and i_n is the noise current[3]. Because our device possesses a broadband frequency response (Figure 4f) which was measured using a lock-in amplifier, D^* of 1.56×10^{13} cm Hz$^{1/2}$ W^{-1} can be obtained at f = 1 kHz.

S13: Temporal response of WSe_2-ZnO LJFETs with relatively small dimensions

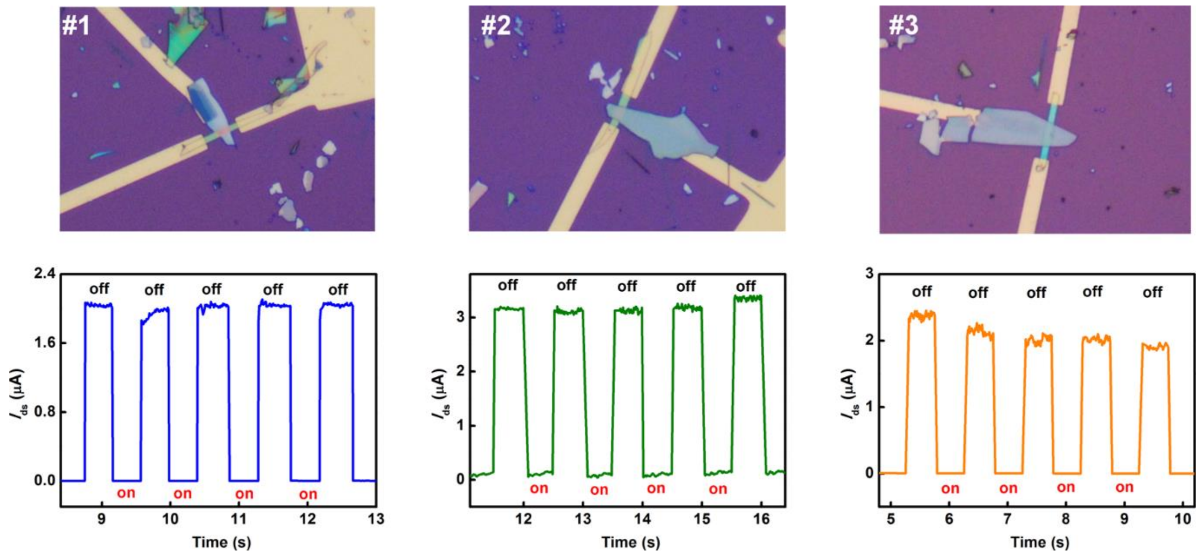

Figure S13. Temporal response of the other three WSe_2-ZnO LJFETs with relatively small dimensions under 637 nm illumination. In order to transfer the WSe_2 nanosheet onto the ZnO channel with a high success rate, the channel length of ZnO is set to be greater than or equal to 9 μm. The channel length (width) of device #1 is ~11 (1.9) μm. The channel length (width) of device #2 is ~9 (2.3) μm. The channel length (width) of device #3 is ~18 (1.8) μm.

S14: AFM measurements of GaSe-ZnO LJFET

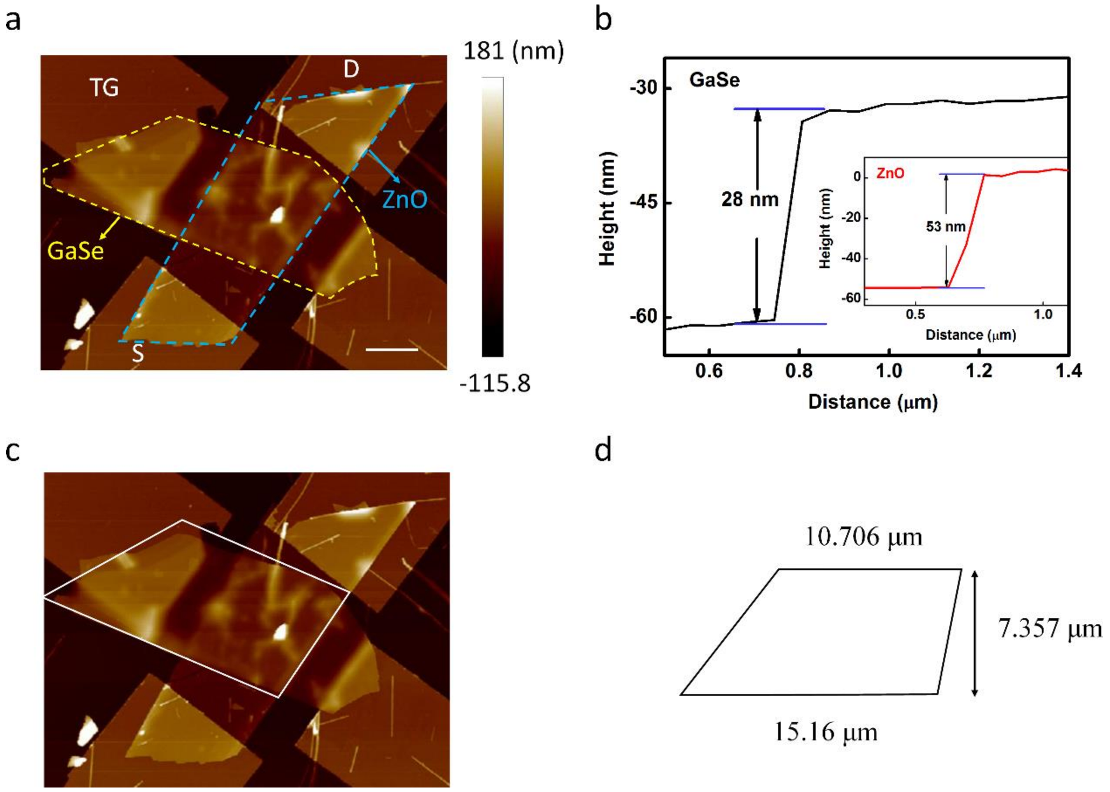

Figure S14. (**a**) AFM image of the GaSe-ZnO LJFET. Scale bar, 3 μm. (**b**) Height profiles of the GaSe nanosheet (black line) and the ZnO belt (red line). (**c**) The effective photosensitive area is indicated by a trapezoid. (**d**) Size of trapezoid used for calculation of effective photosensitive area (95.15 μm^2).

S15: I_{ds}-V_{bg} and I_{ds}-V_{ds} characteristics of bare GaSe transistor

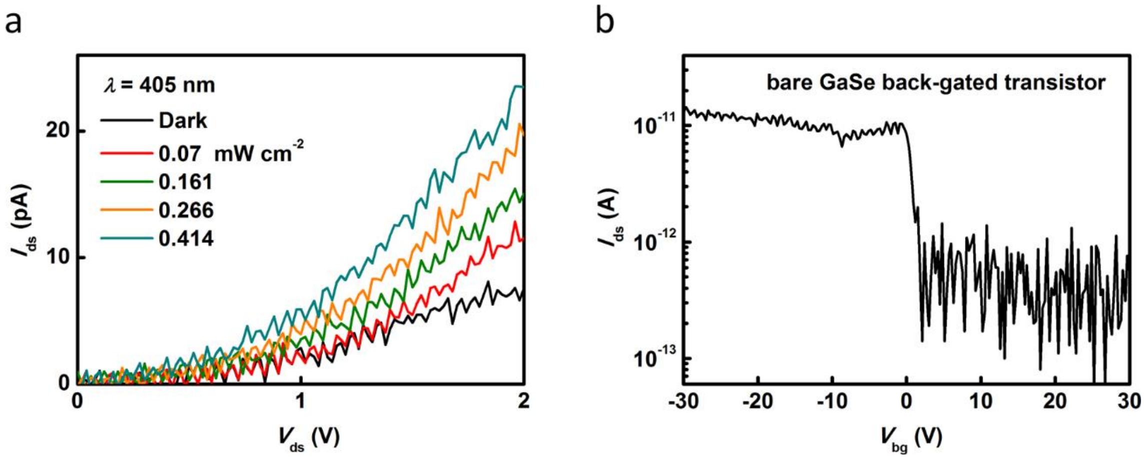

Figure S15. (**a**) I_{ds}-V_{ds} curves of a bare GaSe transistor with variable light power intensity (405 nm) at V_{bg} = 0 V. The voltage is applied to the two side top-gate electrodes which are underneath the GaSe nanosheet (see Figure S14a). The GaSe-ZnO LJFET provides a better performance than the bare GaSe device. (**b**) I_{ds}-V_{bg} curve of a bare GaSe transistor in the dark. The p-type GaSe transistor has a normally off characteristic and does not conduct well both in the accumulation and depletion regimes.

S16: Response time of other GaSe-ZnO LJFETs

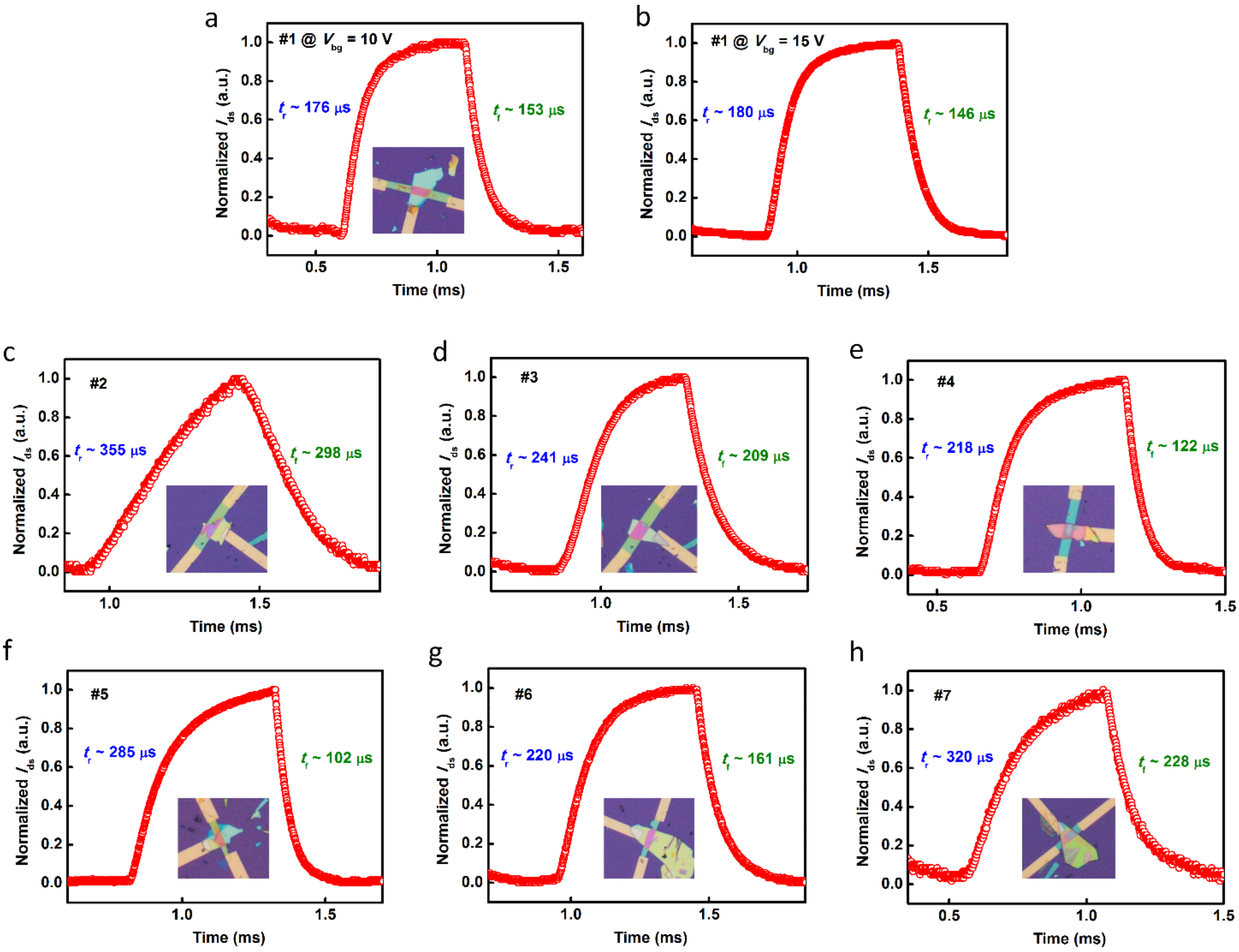

Figure S16. Response time of other GaSe-ZnO LJFETs (405 nm). The insets are the optical photographs of the devices. The measurements were conducted at V_{bg} = 15 V except for (a), which is 10 V.

Considering that the GaSe nanosheet does not conduct well even in the accumulation regime (see Figure S15b), the function of V_{bg} is only to modulate the density of electrons in ZnO for the GaSe-ZnO LJFET. Figure S16a and 16b give the temporal response of device #1 measured at V_{bg} of 10 V and 15 V, respectively. It can be seen that the variation of both the rise and fall time is within 7 μs (~4% change). Therefore, the effect of V_{bg} on the response time (hundreds of microseconds) is negligible.

Figure S16c and 16d give the temporal response of device #2 and device #3, respectively. It should be noted that these two devices use the same ZnO channel but have different GaSe

nanosheets (see the insets). To study the effect of quality of photosensitive material on response time, the same channel was used to exclude the possible effect of different ZnO channels. A large variation of ~100 μs (~30% change) is observed in both rise and fall time. For the device fabrication, we used PDMS to take GaSe nanosheet #2 off and put GaSe nanosheet #3 onto the ZnO channel. Although we cannot acquire the GaSe nanosheets with the same morphology (area and thickness) using mechanical exfoliation, the results still can support the view that the quality of photosensitive material has a great impact on the response time.

Table S1 Response time of typical bare GaSe and WSe_2 photodetectors

Material	Response time	Reference
Mechanically exfoliated few-layer GaSe	20 ms	[4]
Mechanically exfoliated few-layer GaSe	270 μs	[5]
CVD-grown monolayer WSe_2	23 ms	[6]
Mechanically exfoliated 3L WSe_2	10 μs	[7]
CVD-grown 1L WSe_2	>1 ms	[8]

Additionally, the response time of all the GaSe-ZnO LJFETs (#1-#7) is basically at the level of hundreds of microseconds which is longer than that of WSe_2-ZnO LJFET. Many researchers have studied the photoresponse of bare GaSe and WSe_2 detectors. It can be seen from Table S1 that different response times have been reported even for the same material. The difference in photoelectric conversion efficiency and defect states could be the reason for these varying results. Therefore, in the LJFET, the type of photosensitive material also has a great impact on the response time.

References

[1] Z. Wang, R. Yu, X. Wang, W. Wu, Z. L. Wang, *Adv. Mater.* **2016**, *28*, 6880.
[2] M.-H. Doan, Y. Jin, S. Adhikari, S. Lee, J. Zhao, S. C. Lim, Y. H. Lee, *ACS Nano* **2017**, *11*, 3832.
[3] X. Gong, M. Tong, Y. Xia, W. Cai, J. S. Moon, Y. Cao, G. Yu, C.-L. Shieh, B. Nilsson, A. J. Heeger, *Science* **2009**, *325*, 1665.
[4] P. Hu, Z. Wen, L. Wang, P. Tan, K. Xiao, *ACS Nano* **2012**, *6*, 5988.
[5] Y. Cao, K. Cai, P. Hu, L. Zhao, T. Yan, W. Luo, X. Zhang, X. Wu, K. Wang, H. Zheng, *Sci. Rep.* **2015**, *5*, 8130.
[6] W. Zhang, M.-H. Chiu, C.-H. Chen, W. Chen, L.-J. Li, A. T. S. Wee, *ACS Nano* **2014**, *8*, 8653.
[7] N. R. Pradhan, J. Ludwig, Z. Lu, D. Rhodes, M. M. Bishop, K. Thirunavukkuarasu, S. A. McGill, D. Smirnov, L. Balicas, *ACS Appl. Mater. Interfaces* **2015**, *7*, 12080.
[8] J. Chen, B. Liu, Y. Liu, W. Tang, C. T. Nai, L. Li, J. Zheng, L. Gao, Y. Zheng, H. S. Shin, H. Y. Jeong, K. P. Loh, *Adv. Mater.* **2015**, *27*, 6722.
